# Four-week rapamycin treatment improves muscular dystrophy in a fukutin-deficient mouse model of dystroglycanopathy

**DOI:** 10.1186/s13395-016-0091-9

**Published:** 2016-06-02

**Authors:** Steven J. Foltz, Junna Luan, Jarrod A. Call, Ankit Patel, Kristen B. Peissig, Marisa J. Fortunato, Aaron M. Beedle

**Affiliations:** Department of Pharmaceutical and Biomedical Sciences, University of Georgia, 240 W. Green St., Athens, GA 30602 USA; Department of Kinesiology, University of Georgia, Athens, GA 30602 USA; Regenerative Bioscience Center, University of Georgia, Athens, GA 30602 USA

**Keywords:** Dystroglycan, Fukutin, Mammalian target of rapamycin (mTOR), Muscular dystrophy, Rapamycin, Skeletal muscle

## Abstract

**Background:**

Secondary dystroglycanopathies are a subset of muscular dystrophy caused by abnormal glycosylation of α-dystroglycan (αDG). Loss of αDG functional glycosylation prevents it from binding to laminin and other extracellular matrix receptors, causing muscular dystrophy. Mutations in a number of genes, including *FKTN* (fukutin), disrupt αDG glycosylation.

**Methods:**

We analyzed conditional *Fktn* knockout (*Fktn* KO) muscle for levels of mTOR signaling pathway proteins by Western blot. Two cohorts of Myf5-cre/*Fktn* KO mice were treated with the mammalian target of rapamycin (mTOR) inhibitor rapamycin (RAPA) for 4 weeks and evaluated for changes in functional and histopathological features.

**Results:**

Muscle from 17- to 25-week-old fukutin-deficient mice has activated mTOR signaling. However, in tamoxifen-inducible *Fktn* KO mice, factors related to Akt/mTOR signaling were unchanged before the onset of dystrophic pathology, suggesting that Akt/mTOR signaling pathway abnormalities occur after the onset of disease pathology and are not causative in early dystroglycanopathy development. To determine any pharmacological benefit of targeting mTOR signaling, we administered RAPA daily for 4 weeks to Myf5/*Fktn* KO mice to inhibit mTORC1. RAPA treatment reduced fibrosis, inflammation, activity-induced damage, and central nucleation, and increased muscle fiber size in Myf5/*Fktn* KO mice compared to controls. RAPA-treated KO mice also produced significantly higher torque at the conclusion of dosing.

**Conclusions:**

These findings validate a misregulation of mTOR signaling in dystrophic dystroglycanopathy skeletal muscle and suggest that such signaling molecules may be relevant targets to delay and/or reduce disease burden in dystrophic patients.

**Electronic supplementary material:**

The online version of this article (doi:10.1186/s13395-016-0091-9) contains supplementary material, which is available to authorized users.

## Background

The dystrophin-glycoprotein complex (DGC) provides a critical link between the extracellular matrix and the intracellular cytoskeleton to enhance cell membrane stability [1–3]. α-Dystroglycan (αDG) is an extracellular protein within the DGC that acts as a receptor for laminin and other matrix proteins, but it requires the presence of a rare *O*-mannose glycan structure for this function [1, 4–8]. Disruption of αDG *O-*mannose glycosylation impairs its laminin-binding activity causing a group of diseases known collectively as secondary dystroglycanopathies, which are characterized by progressive muscle pathology along with variable involvement of the brain and eyes [9]. To date, mutations in at least 15 genes, including *FKTN*, encoding fukutin, have been identified as causative for secondary dystroglycanopathies [10–13]. Recently, fukutin has been directly implicated in the modification of the core M3 *O*-mannose structure on αDG as a ribitol-5-phosphate transferase that acts upstream of functional modification by like-acetylglucosaminyl transferase (LARGE) [14]. Despite these advances, current therapeutic strategies for dystroglycanopathies are limited, and they remain without treatment or cure.

Disease pathology in DGC-related muscular dystrophies has been attributed to increased susceptibility of the sarcolemma to contraction-induced damage following disruption of αDG-laminin binding, leading to cycles of muscle degeneration and regeneration [2]. Dystrophic muscles lose regenerative capacity over time and muscle fibers are gradually replaced with fibrotic or fatty tissues [15–17]. However, recent evidence suggests that loss of dystroglycan functional glycosylation induces developmental and regeneration-specific defects that may also contribute to disease severity [18–22].

To date, signaling studies in models of dystroglycanopathy have been limited; however, disruption of αDG-laminin interactions might result in downstream signaling consequences. Antibody-mediated laminin detachment has been shown to dysregulate the serine/threonine kinase Akt (protein kinase B), leading to increased apoptosis in cultured myotubes [23]. While best known for its role in cell survival, Akt is also critical for a number of physiological processes in the muscle and has been implicated in the progression of dystrophy in dystrophin-deficient mice [24–26]. Akt is activated through phosphorylation at threonine 308 (T308) by phosphoinositide-dependent kinase 1 (PDK1) and at serine 473 by phosphoinositide-dependent kinase 2 (PDK2). More recently, the elusive PDK2 has been identified as a rapamycin-insensitive complex of mammalian target of rapamycin (mTOR), known as mTOR complex 2 (mTORC2) [27]. Activation of Akt leads to the downstream activation of mTOR complex 1 (mTORC1), which stimulates muscle growth and hypertrophy through increased protein synthesis [28, 29]. Interestingly, mTOR inhibition decreased muscle necrosis and effector T cell infiltration in the mdx mouse, a mild model of Duchenne and Becker muscular dystrophies (DMD/BMD) [30]. Importantly, the muscles from human dystroglycanopathy patients and animal models of disease demonstrate a marked variability in fiber size within a given tissue, often displaying abnormally high populations of both hypertrophic and atrophic cells [18, 31]. These findings suggest that abnormal regulation of cell growth pathways may be a factor contributing to disease pathology. Altogether, manipulation of the molecular mechanisms controlling cell growth and viability might offer an avenue towards the amelioration of dystrophic pathology. Indeed, previous studies have indicated the therapeutic benefit of engaging cell survival signaling [32] or modulating cellular processes involved in cell size, including protein synthesis (through mTOR) and autophagy in other types of muscular dystrophy [33, 34].

In the present study, we show that 17–25-week-old (hereafter, “aged”) *Fktn*-deficient dystroglycanopathy mice with later-stage muscular dystrophy have increased activation of mTOR. However, induction of *Fktn* loss post-development (in 6-week-old mice) failed to change activation status of signaling proteins involved in the mTOR pathway prior to the onset of muscle pathology, indicating that mTOR activation may be a byproduct of the disease state. To better understand whether this change corresponds to pathogenic or compensatory processes in dystroglycanopathy muscle, we investigated the ability of the mTOR inhibitor rapamycin (RAPA) to alter dystrophic pathology. Daily oral dosing of RAPA from 8 to 12 weeks of age reduced histopathology, including proportions of centrally nucleated (CN) muscle fibers, and protected against increased serum creatine kinase (CK) levels following a damaging downhill treadmill run in Myf5/*Fktn* knockout (KO) mice. Ankle dorsiflexors [tibialis anterior (TA), extensor digitorum longus (EDL), and extensor hallucis longus muscles] of RAPA-treated KO mice also produced significantly higher torque post- vs. pre-study, in contrast to untreated KO mice. Immunofluorescent analysis of iliopsoas after completion of the 4-week RAPA study demonstrated mTOR activation (determined by pS6 localization) in both muscle and non-muscle compartments of dystrophic tissue. However, pS6 levels correlated closely with levels of fibrosis in VEH- but not RAPA-treated KO mice. Biochemical analysis revealed increased levels of proteins involved in autophagosome formation in untreated KO mice which were partially reduced following 4 weeks of RAPA treatment. Overall, our data suggest that manipulations in the mTOR pathway may have potential therapeutic benefit. Future studies will be important to define the best pharmacological agents and molecular targets in the mTOR pathway for skeletal muscle improvements in dystroglycanopathies.

## Methods

### Antibodies

The following primary antibodies used in this study were purchased from commercial suppliers: rabbit anti-Akt, p-Akt (S473 and T308), S6, p-S6 (S235/236), p-mTOR (S2448), mTOR, Beclin-1, LC3B, glyceraldehyde 3-phosphate dehydrogenase (GAPDH), and mouse anti-S6 from Cell Signaling (cat# 4691, 4060, 2965, 2217, 4858 or 2211, 5536, 2983, 3738, 2775, 5174, 2317); rabbit anti-Vps15 (A302-571A) from Bethyl Laboratories; rat anti-perlecan from Millipore (MAB1948P); rat anti-CD11b from Fisher (BD Biosciences, BDB550282); dystrophin (MANDYS16) and embryonic myosin heavy chain (eMHC, F1.652) from the Developmental Studies Hybridoma Bank (DSHB); and rabbit anti-collagen VI (ColVI, 70R-CR009x) from Fitzgerald Industries. Antibodies detecting functionally glycosylated αDG (IIH6) and β-dystroglycan protein (βDG, 7D11) have been described previously [1, 35] and were a gift from Dr. Kevin Campbell (U. Iowa) or purchased from DSHB. αDG-core antibodies (45-3, 5-2) were reported recently [36]. Secondary antibodies conjugated to horseradish peroxidase or Alexa Fluor® 488 or 546 were purchased from Millipore, Jackson ImmunoResearch, or Life Technologies.

### Mice

All mouse husbandry and experimental procedures were approved by the University of Georgia Institutional Animal Care and Usage Committee under Animal Use Protocols A2010 08-153 and A2013 07-016 (Beedle). Mice were maintained on a 12:12 h light:dark cycle. Earclips were taken for identification and genotyping. Myf5/*Fktn* conditional KO and Tam/*Fktn* inducible KO mice have been described previously [18, 19]. Female mice homozygous for loxP-flanked (floxed) *Fktn* exon 2 (*Fktn*^L/L^) were crossed to male mice heterozygous for the floxed *Fktn* allele and hemizygous for Myf5-driven Cre-recombinase (*Myf5*^Cre/+^, *Fktn*^L/+^) to generate developmental skeletal muscle knockout of *Fktn* (Myf5^Cre/+^, *Fktn*^L/L^, called Myf5/*Fktn* KO). Whole animal tamoxifen-inducible KO mice (driven by the CAGGCre-ER promoter; Jackson Laboratories, strain #004682) were generated by crossing Tg^Cre-esr1^/^+^, *Fktn*^+/−^ and floxed *Fktn* (*Fktn*^L/L^) mice to give Tg^Cre-esr1^/^+^, *Fktn*^L/−^ progeny (Tam iKO). Due to the incorporation of the null allele, tamoxifen-induced Cre-recombination is required at only one allele to guarantee *Fktn* loss. Both female and male knockout and littermate mice were used for all studies with no preference or special consideration for sex. All study mice, except for the daily rapamycin dosing study with treadmill endpoint, were fasted for 12 h (over the light cycle) prior to tissue collection. For mice in the daily rapamycin dosing study with muscle torque endpoint, the fast was started in the evening following muscle torque measurements for euthanization and tissue collection the following morning.

### Tamoxifen dosing

Tamoxifen (Sigma-Aldrich or Cayman Chemical) was dissolved in 200 proof ethanol and diluted in sunflower seed oil (Sigma-Aldrich) heated to 70 °C. Mice were given two 10 mg doses of tamoxifen by gavage 2 days apart at 6 weeks of age and one additional dose the week of tissue collection. A total of 18 mice were dosed for the study: nine Tam iKO and nine Tam littermate control (LC; Tg^+/+^, *Fktn*^L/−^; Tg^+/+^, *Fktn*^L/+^; or Tg^Cre-esr1^/^+^, *Fktn*^L/+^).

### mTOR pathway pharmacological dosing

Ten milligrams per milliliter stock solutions of rapamycin (RAPA, Calbiochem or VWR Scientific) were made in DMSO (Sigma-Aldrich) and stored at −20 °C. A working solution of 0.5 mg/ml was made by 1:20 dilution in 0.9 % saline. Five percent DMSO in 0.9 % saline was dosed to vehicle control mice (VEH). Insulin (Sigma-Aldrich) was stored as a 7.5 mg/ml stock in 1 % acetic acid and was diluted 1:10 in 0.9 % saline to provide a working solution of 0.75 mg/ml (0.1 % acetic acid in 0.9 % saline was used as the vehicle control). For single dose studies, mice were fasted for a total of 12 h and given vehicle or insulin by intraperitoneal injection at a dose of 0.1 U/g 30 min prior to sacrifice or given vehicle or RAPA (2 mg/kg) by gavage 6 h prior to dissection. A total of five Myf5/*Fktn* KO mice were dosed with vehicle, five with rapamycin, and five with insulin. A total of four littermates were dosed with vehicle, five with rapamycin, and six with insulin.

Daily study mice were administered vehicle (VEH LC or KO) or 2 mg/kg RAPA (RAPA LC or KO) by oral gavage once a day for 4 weeks. In the first daily dosing study (with muscle torque endpoint), five mice were used in each group. In the second daily study (with treadmill endpoint), eight and seven Myf5/*Fktn* KO mice were dosed with RAPA and vehicle, respectively, and six RAPA-treated and six vehicle-treated littermate controls (LC; *Myf5*^Cre/+^, *Fktn*^L/+^; *Myf5*^+/+^, *Fktn*^L/L^; or *Myf5*^+/+^, *Fktn*^L/+^) were used. One RAPA KO met statistical criterion for exclusion and was therefore not included in the final dataset (see statistics).

### Physiological measurements

Mice in the daily rapamycin treatment study were analyzed for serum creatine kinase levels before and after a downhill exhaustion treadmill run. Mice were equilibrated to a four-lane mouse treadmill (Accuscan) at a −10° angle for two 30-min session (25 min at 0 m/min, 5 min at 3 m/min) on days 1 and 2 after the last RAPA or VEH dose. Blood was collected from the ventral tail artery of study mice after the second equilibration session (“pre-exhaustion bleed”). On the third day post-dosing, mice were run using the downhill exhaustion protocol (warm-up 5 min at 3 m/min; downhill exhaustion run 5 min at 10 m/min, 5 min at 15 m/min, 5 min at 20 m/min, and up to 15 min at 25 m/min) [18]. When mice remained on the shock pad for ten consecutive seconds, exhaustion time was recorded and mice were removed from the treadmill. Treadmill run distances for Myf5/*Fktn* KO mice are reported as a normalized comparison to distances of cage- and/or age-matched LC mice running a simultaneous exhaustion protocol. Two hours after the run end, a post-treadmill blood sample was collected and mice were sacrificed for tissue collection. For creatine kinase measurements, serum was diluted 1:10 in ultrapure water and added to creatine kinase reagent (StanBio) according to the manufacturer’s protocol. The resulting mixture was assayed for CK activity using a Synergy 2 microplate reader (BioTek Instruments).

Torque was measured from the ankle dorsiflexors of mice as previously described [37, 38]. Mice were anesthetized with 1.5 % isoflurane-mixed oxygen, hair was removed from the left lower hind limb, and the foot was attached to a servomotor for torque measurement (Aurora Scientific, Aurora Canada). Muscle contraction was stimulated using Pt-Ir needle electrodes inserted percutaneously adjacent to the peroneal nerve using 1 to 2 mA (stimulator model 701C, Aurora Scientific). Isometric torque was measured in response to 20, 40, 60, 80, 100, 125, 150, and 200 Hz of stimulation frequency. Each mouse in the RAPA daily study with muscle torque testing underwent two separate muscle torque exams, one prior to the onset of RAPA or VEH dosing (the “pre” test) and one on the day following the 28th RAPA or VEH dose (the “post” test). Torque measurements were normalized to body mass (in kilograms) for each mouse to account for size differences between animals.

### Kidney and liver toxicity measurements

Blood urea nitrogen (BUN) and serum alanine transaminase (ALT) were assayed using kits purchased from Arbor Assays and Cayman Chemicals, respectively. For BUN, serum was diluted 1:20 in ultrapure H_2_O and analyzed by colorimetry according to the manufacturer’s protocol on a Spectramax microplate reader using Softmax Pro 5.3 software (Molecular devices). ALT was detected in serum diluted 1:10 in ultrapure H_2_O via consumption of NADH in a coupled reaction. Absorbance measurements were taken at 340 nm (Synergy 2 microplate reader) every minute for 5 min and the average rate of change across a linear range was used to quantitate ALT.

### Succinate dehydrogenase measurement

TA muscles were homogenized on ice with a glass-on-glass dounce homogenizer in 2 ml of 33 mM PO_4_^3−^ buffer. Samples were aliquoted and taken through 3 cycles of freezing and thawing before use. A method for spectrophotometric determination of succinate dehydrogenase [39] was adapted for the microplate. Triplicate 4 or 8 μl volumes of tissue homogenate were incubated with 10 μl of 0.5 M sodium succinate for 2 min at 30 °C. Ten microliters of sodium cyanide was mixed into samples followed by 300 μl cytochrome C working reagent (0.1 M cytochrome C from equine heart in 0.17 M PO_4_^3−^ buffer, 0.004 M AlCl_3_/CaCl_2_, and H_2_O mixed 2.5:1:7.5) immediately prior to reading (Spectramax microplate reader). Reduction of cytochrome C was assessed by kinetic absorbance measurements at 550 nm. Data are presented as micromole cytochrome c reduced per minute per mg tissue mass.

### Histology and microscopy

Seven-micron tissue cryosections were stained by hematoxylin and eosin (H&E) according to standard protocols. Sections were processed for secondary immunofluorescence (IF) as described previously [18, 19, 40]. H&E- or immunofluorescent-stained skeletal muscle sections were imaged using an X71 inverted epifluorescent microscope with a Peltier element-cooled 12.8MP DP72 CCD camera and CellSens software (Olympus).

For analysis of eMHC, αDG glycosylation (IIH6) and central nucleation, tiled ×20 images were taken across each entire muscle section and aligned to compile entire section maps in Photoshop (Adobe), then positive and negative fibers were counted manually using ImagePro Express (MediaCybernetics). Data are reported as percentages obtained by dividing the number of positive fibers by the total number of muscle fibers in an entire muscle section. Tissue maps from this analysis were also used for measurement of muscle fiber minimum diameter. Perimeters of individual muscle fibers were traced in a semi-automated fashion using a combination of manual segmentation (dark objects) and manual polygon selection in ImagePro Premier v9.1 (Media Cybernetics), which calculated minimum diameter according to a reference distance (scale bar). Area values were sorted according to size, grouped into bins of 2.5 μm (estimated from the formula $h=\frac{3.5\sigma }{n^{1/3}}$, where *h* is the optimized bin size, *σ* is the standard deviation, and *n* is the number of observations), up to a maximum of 30 μm and plotted as a histogram. Analyses were performed blinded to experimental group and study design.

Sections incubated with anti-collagen VI, anti-CD11b, or anti-pS6 were imaged and mapped as above. Whole section maps were analyzed for fluorescent area using Fiji (ImageJ, [41]) software. Briefly, a region of interest was drawn to encircle the entire muscle section. The number of pixels/section was quantified and a color threshold was set. Pixels at brightness above the color threshold were considered positive for collagen VI, CD11b, or pS6; these pixels were selected and counted. Data are expressed proportion of area, which is the number of positive pixels/total number of pixels in the section for each map.

### Western blotting

Pooled hind limb muscles (soleus, gastrocnemius, tibialis anterior [TA], hamstring, quadriceps, and gluteus) or isolated quadriceps from individual mice were homogenized and solubilized in buffer containing 50 mM Tris (pH 7.4), 150 mM NaCl, 1 % Triton-X, and a homemade cocktail of phosphatase and protease inhibitors: pepstatin A, 0.6 μg/ml; aprotinin, 0.5 μg/ml; leupeptin, 0.5 μg/ml; phenylmethanesufonylfluoride, 0.1 mM; benzamidine, 0.75 mM; calpain I inhibitor, 2 μM; calpeptin, 2 μM; NaF, 50 mM; NaPP_i_, 10 mM; and β-glycerophosphate, 10 mM. Initial studies included NaVO_4_ for inhibition of tyrosine and alkaline phosphatases. However, there was marked sample precipitation over time, so NaVO_4_ was excluded from all samples reported here. Insoluble material was pelleted at ×142,000*g* for removal, and the resulting supernatant was filtered through cheesecloth. Protein concentrations were determined using a modified DC protein assay (Bio-Rad) with a SpectraMax microplate reader (Molecular Devices).

Solubilized skeletal muscle protein samples (500 μg) were loaded onto 3–15 % gradient gels for SDS-PAGE. Protein was transferred to PVDF (Millipore), blocked with 1 % milk in Tris-buffered saline with 0.1 % Tween-20 (TBS-T), and probed with primary antibody overnight, as described previously [18, 40]. Secondary antibodies conjugated to horseradish peroxidase were used, and blots were imaged with West Pico or West Dura chemiluminescent reagent (Pierce) and the Fluorchem HD2 digital imager (Protein Simple). All study mice (*n* = 4–8 per group) from each group were tested by Western blot with a minimum of two technical replicates. One sample (VEH LC) in the daily RAPA dosing study was unfit for analysis by Western blot and was excluded from those data sets.

### Cytokine/chemokine multiplex assay

Transforming growth factor-β (TGF-β, TGFBMAG-64 K-01) or mouse cytokine/chemokine (MCYTOMAG-70k: interleukin-1β, monocyte chemotactic protein-1, and tumor necrosis factor-α) magnetic bead kits were purchased from Millipore for analysis via the Luminex xMAP platform. Solubilized protein (50 μg) or diluted serum samples (1:30, TGF-β; 1:4 cytokine/chemokine multiplex) were loaded onto a pre-wet 96-well plate and incubated with antibody-conjugated magnetic beads overnight at 4 °C. Beads were pelleted using a handheld magnet, samples were decanted, and pelleted beads were resuspended in the presence of biotinylated detection antibodies. Finally, the beads were treated with streptavidin-conjugated phycoerythrin and imaged with the MAGPIX (Luminex Corp.) using xPONENT 4.2 software. Median fluorescent intensity (MFI) for each sample was normalized as fold increase over background MFI.

### Graphing, data analysis, and statistics

Band densitometry for Western blot was measured using AlphaView 3.0 software (Protein Simple). Band intensities of a given blot were set as a proportion of the maximum intensity (equal to 1), and these values for the protein of interest were normalized to the corresponding value of the total protein content for phopho-epitopes of signaling proteins or to loading control (GAPDH) for all others.

Data are plotted as scatter plots (each individual data point represents one study mouse) with the group mean and standard error of the mean using Prism 5 (GraphPad). As a number of plots contained possible outlier samples, a Dixon’s *q*-test [42, 43] was applied to every data set with a critical value of *α* = 0.05 to identify true outliers. A number of datasets contained an outlier in one of the study groups. For the most part, the individual outlier mouse was not consistently an outlier across datasets; therefore, no action was taken in these cases. However, one mouse in the daily dosing study was a clear outlier in the majority of its datasets. Therefore, this mouse was excluded from reported data. Differences between study groups were determined by two-tailed Student’s *t* test or two-way analysis of variance (ANOVA) with Bonferroni’s post-test in Prism 5. The interaction (e.g., Drug*Genotype) *p* value from two-way ANOVA analysis is reported on all figures, and individual main effects (e.g., drug, genotype) are reported only in the absence of a significant interaction between variables. Statistical significance between groups is denoted by *p < 0.05; **p < 0.01; ***p < 0.001. A significant difference between one group and two or more other groups is represented on plots by a line originating from the first group, with downward ticks indicating each group from which it differed. Asterisks above downward ticks indicate significance level. Fiber size distributions were compared by two-way ANOVA at each bin size (Prism 5, GraphPad). A significant interaction (Drug*Genotype *p* < 0.05) is marked by an asterisk and a significant genotype (*p* < 0.05) effect is marked by a dagger sign. Correlations were determined by Pearson (Gaussian) or Spearman (non-parametric) tests.

## Results

### The Akt/mTOR signaling axis is altered in dystroglycanopathy muscle

**Fig. 1:**
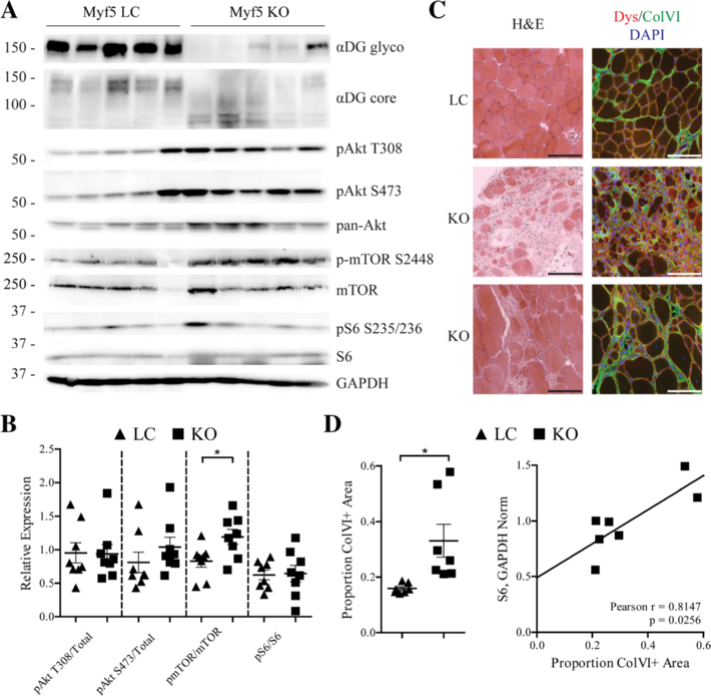
mTOR is activated in aged, fasted Myf5/*Fktn* KO muscle. **a** Western blot analysis of solubilized protein from the hind limb muscle of Myf5/*Fktn* LC and KO mice. **b** Quantification of Akt phosphorylation at T308 and S473, mTOR phosphorylation at S2448, S6 phosphorylation at S235/236, relative to total Akt, mTOR, and S6 protein, respectively, as a measure of protein activation. mTOR phosphorylation at S2448 normalized to total mTOR is significantly increased in KO muscle. Two-tailed Student’s *t* test; *p < 0.05. **c** Representative images of iliopsoas muscle from aged LC or KO mice. H&E staining and dystrophin (*red*), ColVI (*green*), and DAPI (*blue*) immunofluorescence are shown. Scale bar 100 μm. (**d**, *Left*) Quantification of ColVI in iliopsoas of aged mice reveals significantly increased collagen content in KO muscle. Two-tailed Student’s *t* test; *p < 0.05. (**d**, *Right*) Total S6 protein correlates with ColVI quantity in KO muscle (Pearson *r* = 0.8147; *p* = 0.0256). *n* = 8 Myf5/*Fktn* LC and 8 Myf5/*Fktn* KO mice (*n* = 7 per group for ColVI analysis due to tissue artifacts)

Abnormal intracellular signaling has been reported in various forms of muscular dystrophy, including other DGC-related diseases (reviewed in [44]), but not in dystroglycanopathy. To determine whether Akt/mTOR signaling is altered in dystroglycanopathy mice, we examined the activation status of pathway proteins in fasted Myf5/*Fktn* KO muscle from mice aged 17–25 weeks, an age range with substantive remodeling of the muscle compartment. Myf5/*Fktn* KO mice were selected for this study because (1) *Fktn*-deficiency is the most common form of congenital dystroglycanopathy; (2) this conditional knockout model bypasses embryonic lethality associated with total *Fktn* loss; and (3) *Fktn* excision is initiated during muscle development causing moderate to severe dystroglycanopathy, similar to the phenotypic spectrum observed in human patients. As expected, Myf5/*Fktn* KO mice had significantly less functional αDG glycosylation in solubilized hind limb skeletal muscle than age-matched LC mice (Fig. 1a). Akt activation status was not different in Myf5/*Fktn* KO compared to LC mice using either T308 or S473 normalized to total Akt protein (Fig. 1a, b). In contrast, the activated pool of mTOR, the kinase subunit of mTORC1 and mTORC2, was increased (phospho-S2448/total mTOR) (Fig. 1a, b). mTOR serine 2448 is phosphorylated in a feedback loop by mTORC1 substrate S6 kinase 1 [45, 46], indicating elevated mTORC1 activity in the skeletal muscle of Myf5/*Fktn* KO compared to LC. Despite this indication of elevated S6 kinase activity, phosphorylation of its downstream target, translation initiator ribosomal protein S6, was not significantly increased in Myf5/*Fktn* KO vs. LC mice relative to the total S6 protein although total S6 protein was elevated (Fig. 1a, b; Additional file 1). The considerable variability in expression of Akt and S6 proteins in Myf5/*Fktn* KO mice (Additional file 1) may reflect differences in the severity of muscular dystrophy within the aged knockout cohort. Therefore, the iliopsoas muscle was examined histologically for inter-individual disease pathology. The iliopsoas was chosen because it is a proximal muscle more severely affected in Myf5/*Fktn* KO mice and has a relatively small cross-sectional area with less intra-section variability in pathology. As expected, there was an extensive fibrosis (increased collagen VI, ColVI) and variation in muscle fiber size in iliopsoas muscles of the aged Myf5/*Fktn* KO mice, but dystrophy in some mice was more severe than in others (Fig. 1c, d). When total S6 protein expression was plotted versus ColVI fibrosis for individual Myf5/*Fktn* KO study mice, there was a significant positive correlation (Pearson *r* = 0.8147; Fig. 1d). Therefore, some variation in cellular signaling pathways may be explained by a loss of muscle fibers and/or an increase in the fibrotic content of the muscle compartment in late-stage dystrophic Myf5/*Fktn* KO animals.

To determine whether abnormal activation of mTOR in KO muscle is due to a change in its ability to integrate input from external ligands, we probed the pathway by acute activation with insulin in Myf5/*Fktn* KO and LC mice. Twelve-week-old mice were chosen because muscle fibers populate a relatively larger proportion of the tissue compartment in Myf5/*Fktn* KO muscle at this time point. Animals were fasted for a total of 12 h and treated with an insulin challenge or vehicle in the last 30 min. The dystroglycan glycosylation deficiency was confirmed in KO mice and S6 ribosomal protein activation was tested by Western blot as a downstream readout of mTORC1 activity. Insulin significantly induced activation of S6, the target of mTORC1 substrate S6 kinase. Phosphorylation at S235/236 relative to S6 protein was increased in both LC (3.84 ± 1.26-fold) and KO muscle (3.07 ± 0.75-fold) (two-way ANOVA, drug effect *p* = 0.0255). To assess basal levels of mTORC1 activity at this age, a separate cohort of mice was dosed with mTOR inhibitor rapamycin (RAPA) or vehicle 6 h before muscle collection. RAPA reduced the ratio of S6 phosphorylation to S6 protein in fasted littermates (4.71 ± 2.40-fold) and Myf5/*Fktn* KO (7.24 ± 3.55-fold) compared to time-matched vehicle controls (two-way ANOVA drug effect *p* = 0.0147). These results demonstrate that mTORC1 retains the capacity to respond to acute activation or inhibition in Myf5/*Fktn* KO mice.

### mTOR activation is not coincident with loss of functional αDG glycosylation

Although our results indicate misregulation of mTOR signaling in Myf5/*Fktn* KO mice, it is unclear whether this finding results directly from improper glycosylation of αDG or from the progressive dystrophic process. For example, changes in mTOR signaling could arise from any of these possibilities: first, loss of αDG binding to laminin could change the activation status of signaling molecules to directly affect the pathway changes identified here (e.g., [23]). Second, mTOR signaling could be initiated by disease processes contributing to the pathological progression, including muscle regeneration [24, 25, 47], differentiation and growth of replacement fibers [29, 48–50], or fibrosis [51, 52]. Finally, mTOR pathway activation could be a compensatory adaptation by the muscle to mitigate damage burden on the tissue.

**Fig. 2:**
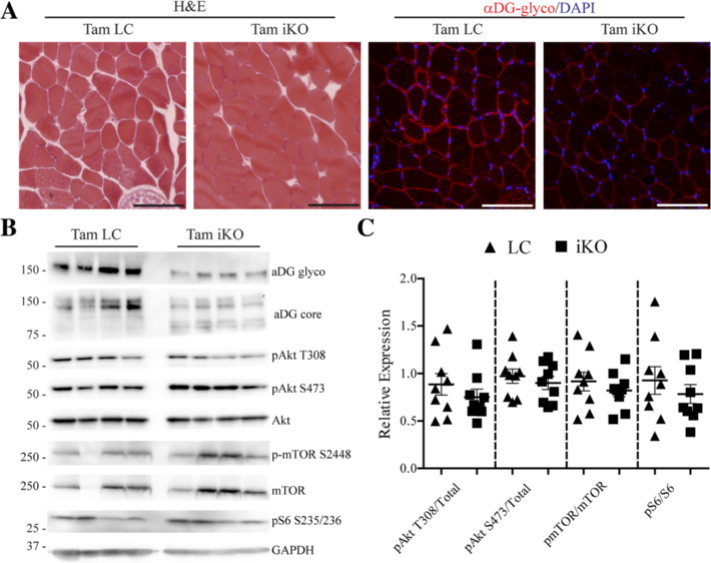
Akt/mTOR signaling is unchanged following loss of αDG glycosylation. **a** Representative images of littermate (Tam LC) or inducible knockout (Tam iKO) iliopsoas. H&E and αDG glyco images are shown. Scale bar = 100 μm. **b** Western blot analysis of solubilized skeletal muscle from hind limbs of Tam LC or Tam iKO mice. **c** Quantification of Akt phosphorylation at T308 and S473, mTOR phosphorylation at S2448, and S6 phosphorylation at S235/236 relative to total Akt, mTOR, and S6 protein, respectively, as a measure of protein activation. Data are presented as mean ± SEM. *n* = 9 per group

To address the first possibility, we employed the whole-body inducible *Fktn*-KO (iKO) mouse to induce *Fktn* exon 2 deletion and subsequent deficiency in αDG glycosylation post-development, a model we reported previously to have a reduction of αDG glycosylation as early as 2.5 weeks and evidence of muscle damage by 8 to 10 weeks post-tamoxifen [18]. Adult mice were dosed with tamoxifen and euthanized (fasted) 5 weeks later to ensure that αDG glycosylation was disrupted, but dystrophic pathology was not yet present. Iliopsoas of tamoxifen-treated iKO mice (Tam iKO) were histologically normal but showed reduced immunoreactivity to IIH6 antibody against the glycosylated epitope of αDG (Fig. 2a); furthermore, decreased αDG glycosylation and a shift to lower molecular weight αDG protein was confirmed by Western blot (Fig. 2b). Analysis of Akt, mTOR, and S6 activation revealed no significant differences between Tam LC and Tam iKO mice (Fig. 2b, c) suggesting that abnormal mTOR signaling is not a direct consequence of αDG hypoglycosylation.

### Four-week daily mTORC1 inhibition improves disease features in dystroglycanopathy mice

These data indicate that mTOR activation in aged Myf5/*Fktn* KO is likely related to the dystrophic phenotype but do not distinguish between potential pathogenic or protective roles for this signal. Although previous studies have demonstrated the ability of rapamycin to reduce the dystrophic phenotype in some models of muscular dystrophy, complete ablation of mTORC1 signaling through knockout of raptor, an obligate member of mTORC1, leads to a dystrophic phenotype in mice [30, 33, 53]. This might indicate that mTORC1 signaling is necessary for the maintenance of normal skeletal muscle but becomes pathogenic under disease conditions. To test whether mTORC1 signaling contributes to pathology in Myf5/*Fktn* KO mice, 8-week-old KO and LC mice were dosed with 2 mg/kg RAPA or vehicle control (VEH) daily for 4 weeks by oral gavage. Although RAPA is approved for clinical use, adverse effects including nephrotoxicity and hepatotoxicity, have been reported [54]. To determine whether the 4-week daily RAPA treatment induced damage to the kidneys or liver, we evaluated blood urea nitrogen (BUN) and serum alanine transaminase (ALT) levels in study mice during the final week of dosing. No effect of RAPA treatment was observed for either analyte: BUN (mg/dL) = 22.39 ± 2.979, VEH LC; 20.37 ± 2.698, RAPA LC; 17.50 ± 3.70, VEH KO; 22.10 ± 2.88, RAPA KO (two-way ANOVA, drug *p* = 0.6812) and ALT (U/L) = 75.73 ± 38.51, VEH LC; 66.30 ± 21.52, RAPA LC; 86.84 ± 36.78, VEH KO; and 40.94 ± 13.83, RAPA KO (two-way ANOVA, drug *p* = 0.3552). Therefore, we have no evidence of treatment-related toxicity in the 4-week 2 mg/kg RAPA dosing regimen to dystroglycanopathy mice.

**Fig. 3:**
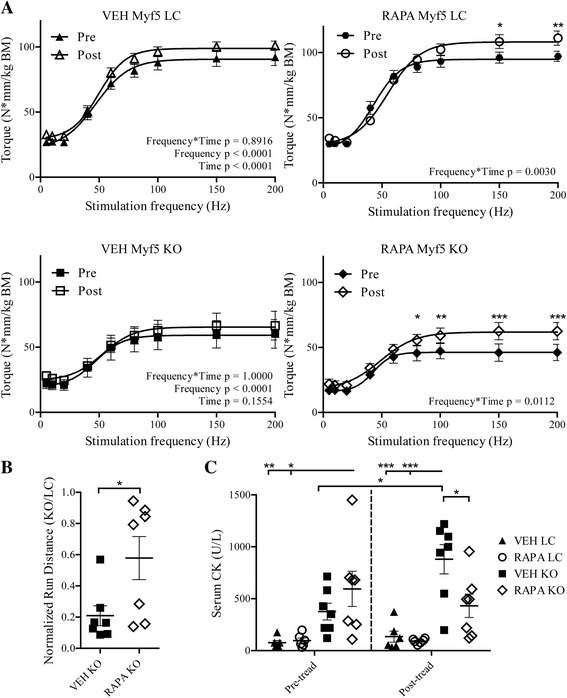
Four-week daily RAPA treatment improves functional outcomes in Myf5/*Fktn* KO mice. **a** Force-frequency curves showing in vivo force measurements taken from tibialis anterior muscles of daily RAPA study mice prior to and at completion of RAPA or VEH dosing. Two-way repeated measures ANOVA with Bonferroni’s post-test. *p < 0.05; **p < 0.01; ***p < 0.001. *n* = 5 mice per group. **b** Comparison of downhill treadmill distances run by VEH and RAPA KO mice. Data are presented as the proportion of KO distance normalized to respective age- and treatment-matched LC mice from the same treadmill run. Two-tailed Student’s *t* test. *p < 0.05. *n* = 7 mice per group. **c** Serum creatine kinase analysis of tail vein bleeds taken from daily RAPA study mice at the end of dosing (*left*, pre-tread) or 2 h after a downhill run to exhaustion (*right*, post-tread). Two-way ANOVA with Bonferroni’s post-test of all pairs. *p < 0.05; **p < 0.01; ***p < 0.001. *n* = 6, VEH and RAPA LC; *n* = 7, VEH and RAPA KO

We employed two separate study designs to enable the analysis of distinct functional outcomes: in the first study, we obtained in vivo torque measurements from ankle dorsiflexors prior to and at the completion of the dosing regimen and isolated single, fasted muscles for biochemical analysis. In the second study, tail vein blood was collected for the determination of serum CK from study mice before and after a downhill treadmill run to exhaustion. Isometric torque measurements were taken across a spectrum of stimulation frequencies (5, 10, 20, 40, 80, 100, 150, and 200 Hz) in order to evaluate contractile ability while still simulating physiologic contraction, which occurs in rodents between 60 and 100 Hz [55]. Data are plotted as a force-frequency curve to provide direct measurement of the frequency-dependence of tetanic contraction and the maximal torque, which is an indirect measure of maximal muscle force. Assessment of resultant force-frequency curves demonstrated an age-related improvement in dorsiflexor torque (pre vs. post) of VEH LC mice that was absent in VEH KO mice, indicating that Myf5/*Fktn* KO mice do not experience muscle strength improvements typical with maturation to adulthood (Fig. 3a). In contrast, there was a significant interaction between frequency and time (pre vs. post) for both RAPA-treated LC and KO mice demonstrating robust gains in isometric torque at several stimulation frequencies (i.e., 150–200 Hz in RAPA LC and 80–200 Hz in RAPA KO) (Fig. 3a). Maximal torque of LC and KO mice receiving RAPA improved by 14.02 ± 4.68 and 14.32 ± 3.66 N*mm/kg body mass, respectively, compared to a modest increase in VEH LC (7.92 ± 3.61) and highly variable changes in VEH KO mice (6.97 ± 12.10 SEM). In the second cohort, 4-week RAPA treatment significantly increased treadmill run distances of Myf5/*Fktn* KO mice normalized to age- and trial-matched LC mice, corroborating the functional improvement seen via muscle force testing (Fig. 3b).

CK values, indicating the presence of muscle damage, were also notably different between RAPA and VEH KO mice. First, basal serum CK (pre-treadmill) in VEH KO mice was not statistically different from LC mice (Fig. 3c), a finding consistent with our previous work demonstrating elevated serum CK levels only in young Myf5/*Fktn* KO mice [18]. This can be explained by the gradual replacement of CK-containing muscle fibers with fibrotic tissue during disease progression, meaning that fewer muscle fibers are available to leak CK to the serum in older mice. However, basal serum CK was increased in RAPA KO mice (Fig. 3c), suggesting a RAPA-induced delay in Myf5/*Fktn* KO pathology. Importantly, serum CK was significantly increased over basal levels by a downhill exhaustion run in VEH, but not RAPA, KO mice, suggesting that the 4-week daily RAPA treatment was protective against muscle injury in Myf5/*Fktn* KO mice (Fig. 3c).

**Fig. 4:**
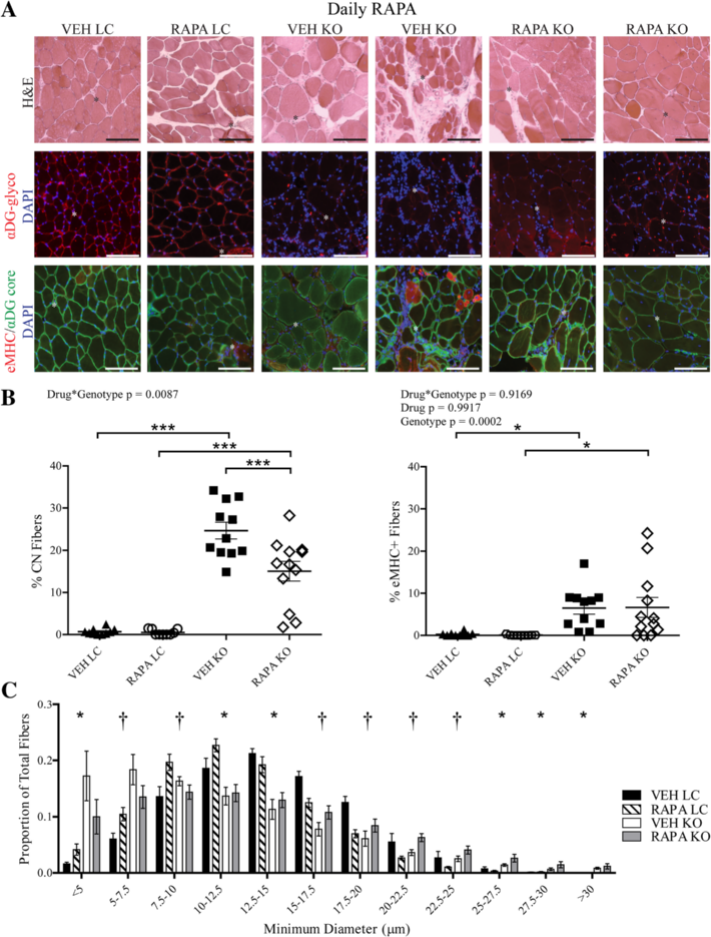
Daily RAPA reduces central nucleation and alters fiber size of Myf5/*Fktn* KO iliopsoas. **a** Images from iliopsoas muscles of VEH- or RAPA-treated LC and KO mice. H&E, functionally glycosylated αDG, and core αDG/embryonic myosin heavy chain (eMHC) are shown. Nuclear DAPI counterstain is shown in blue. Scale bar = 100 μm. *Asterisks* denote identical tissue locations across images. Muscle fiber regeneration as measured historically by (**b**, *left*) central nucleation (CN) or acutely by (**b**, *right*) eMHC. Data are plotted as ((#positive fibers/total fibers)*100). Two-way ANOVA; *p < 0.05; ***p < 0.001. *n* = 10 VEH LC, *n* = 9 RAPA LC, *n* = 12 VEH KO, *n* = 12 RAPA KO. **c** Distribution of muscle fiber minimum diameter from iliopsoas of VEH- and RAPA-treated LC and KO mice. Fibers are grouped into bins of 2.5 μm. Two-way ANOVA performed for each bin. *, Drug*Genotype *p* < 0.05; †, Genotype *p* < 0.05. *n* = 5 VEH LC (tissue artifact), *n* = 5 RAPA LC (tissue artifact), *n* = 7 VEH KO, *n* = 7 RAPA KO

If muscle injury were indeed reduced in RAPA KO mice, regenerative markers might likewise be decreased since regeneration in dystrophic muscle indicates prior degeneration. To evaluate cumulative regeneration across the timeline of the dosing study, we analyzed central nucleation (CN), which persists for several weeks to months following a regeneration event, in muscle fibers of the iliopsoas [56]. RAPA KO mice had significantly fewer centrally nucleated fibers compared to VEH KO mice (Fig. 4a, b). However, this could signify either that RAPA treatment reduces muscle damage or that it inhibits subsequent regeneration. To clarify this point, we assessed levels of embryonic myosin heavy chain (eMHC)-positive fibers, which transiently mark regeneration, in iliopsoas of RAPA-study mice. eMHC-positive fibers were unchanged between VEH and RAPA KO iliopsoas and were increased compared to drug-matched LC iliopsoas (two-way ANOVA, drug *p* = 0.9917, genotype *p* = 0.0002) (Fig. 4a, b). Together, these results indicate that muscles in RAPA KO mice retain regenerative capacity in the short term and support a myoprotective effect of RAPA treatment.

*Fktn*-deficient dystrophic muscle exhibits notable fiber size variability populated by an abundance of both small, atrophic fibers and larger, hypertrophic fibers. Since mTOR is a well-studied regulator of muscle fiber size, we hypothesized that RAPA treatment might alter this distribution in treated mice. Cross-sectional minimum fiber diameters were measured for all muscle fibers in the iliopsoas of RAPA-study mice. Littermate mice show more evenly distributed minimum fiber diameters, with few or no atrophic fibers. In contrast, fibers from both RAPA and VEH KO muscle had significantly higher proportions of small-sized fibers and an expanded proportion of fibers with minimum diameters greater than 25 μm (Fig. 4c). In accordance with this, we observed a significant effect of genotype on fiber size at 6 of the 12 bin sizes. Furthermore, there was a significant Drug*Genotype interaction at the 5–7.5, 7.5–10, 15–17.5, 17.5–20, 20–22.5, and 22.5–25 μm minimum fiber diameter bins. To confirm that differences in fiber size between VEH and RAPA KO mice were not due simply to a reduction in small, regenerating fibers, we further analyzed distributions of regenerated (in KOs only) and non-regenerated muscle fibers separately. We found little difference between the fiber diameter distributions of all fibers compared to non-regenerated fibers only; furthermore, the proportions of regenerated fibers at each bin size were different only at the 20–22.5-μm bin, indicating that changes in the overall fiber size distributions are likely not attributable to differences in the pool of regenerating fibers (Additional file 2). Thus, RAPA shifted fiber size towards larger values in KO muscle but relatively smaller values in LC muscle, further supporting the idea that *Fktn*-deficient muscle has some abnormal, RAPA-sensitive signaling activity.

**Fig. 5:**
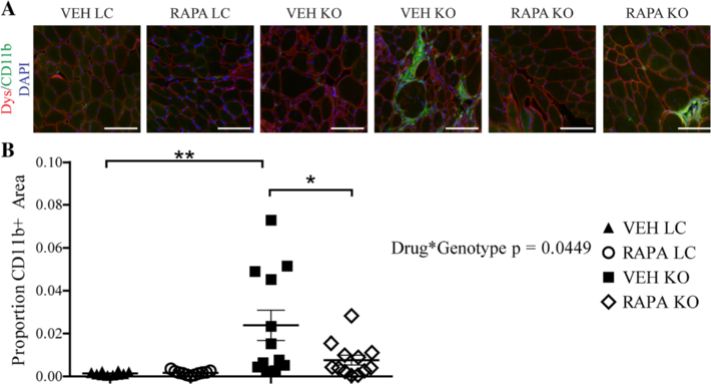
Four-week daily RAPA treatment decreases immune cell infiltration in iliopsoas. **a** Images from transverse sections stained for macrophage marker CD11b (*green*), with dystrophin (*red*) and nuclear DAPI (*blue*) counterstains. Scale bar = 100 μm. **b** Quantification of CD11b-positive areas in iliopsoas of daily study mice. Data are presented as the proportion *green* pixels per tissue/total pixels per tissue. Two-way ANOVA with Bonferroni’s post-test; *p < 0.05; **p < 0.01. *n* = 10 VEH LC, *n* = 11 RAPA LC, *n* = 12 VEH KO, *n* = 12 RAPA KO

Muscle damage is often followed by an inflammatory response involving macrophage infiltration that helps to mediate tissue repair. However, dystrophic muscle experiences repeated cycles of damage and repair with persistence of the inflammatory phenotype [57, 58]. As RAPA treatment appears to offer protection against muscle damage, we hypothesized that localized tissue inflammation might be reduced in RAPA-treated Myf5/*Fktn* KO mice. We incubated iliopsoas muscle sections from study mice with an antibody detecting CD11b, a common marker for mature macrophages that may also be expressed by granulocytes, dendritic cells, and NK cells [59]. Tissues from both LC and KO mice were positive for isolated, “resident” immune cells; however, KO iliopsoas also displayed regions of intense staining likely marking major inflammatory invasion (Fig. 5a). Importantly, RAPA KO mice had significantly less CD11b-positive fluorescence than VEH KO mice, indicating reduced inflammation with RAPA treatment (Fig. 5b). To determine if changes in tissue immune cell content corresponded to altered levels of cytokines in RAPA KO mice, we tested solubilized protein from the quadriceps muscle of study mice for the presence of pro-inflammatory (interleukin-1β, monocyte chemotactic protein-1, tumor necrosis factor α) cytokines in multiplex via MILLIPLEX Map assay. We observed a significant effect of genotype on IL-1β levels when normalized to assay background; however, we noted no differences in other pro-inflammatory cytokines tested (Additional file 3A). Thus, IL-1β appears to be a RAPA-insensitive modulator of inflammation in dystroglycanopathy muscle.

**Fig. 6:**
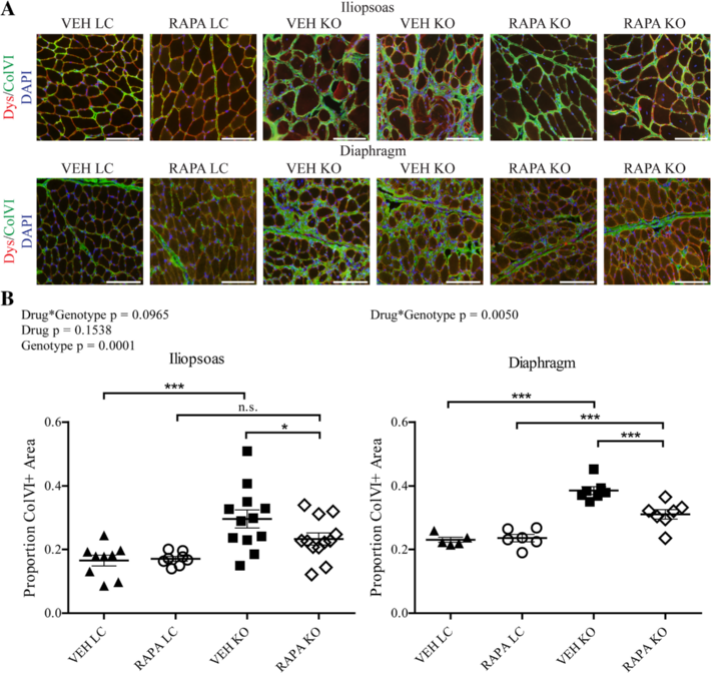
Fibrosis is reduced in iliopsoas and diaphragm of RAPA-treated Myf5/Fktn KO mice. **a** Representative images from iliopsoas (*top*) or diaphragm (*bottom*) of daily study mice stained with dystrophin/ColVI; nuclear DAPI counterstain shown in *blue*. Scale bar = 100 μm **b** Quantification of muscle fibrosis, ColVI-positive area, in iliopsoas (*left*) or diaphragm (*right*) muscles, detected by immunofluorescence and counted as positive (*green*) pixels per tissue/total pixels per tissue. Two-way ANOVA; *p < 0.05; ***p < 0.001. Ilio: *n* = 9 VEH LC, *n* = 8 RAPA LC, *n* = 12 VEH KO, *n* = 12 RAPA KO. Dia: *n* = 5 VEH LC, *n* = 6 RAPA LC, *n* = 7 VEH KO, *n* = 7 RAPA KO

Although the muscle possesses considerable potential for regeneration, dystrophic muscle eventually loses regenerative capacity, due at least in part to chronic inflammatory conditions [58]. Failed muscle regeneration frequently results in the replacement of functional muscle with fibrotic scar tissue, comprised largely of excess extracellular matrix proteins. Consistent with a fibrotic phenotype in *Fktn*-deficient muscle, transforming growth factor-β (TGF-β), a major fibrogenic cytokine, was significantly increased in muscle of KO relative to LC study mice as determined by MILLIPLEX Map analysis, but we did not detect any RAPA-mediated reduction in TGF-β (Additional file 3B). However, extracellular matrix protein ColVI, which accumulates abnormally in dystrophic muscle [60, 61], was increased in histological sections of iliopsoas muscle of VEH KO mice, demonstrating substantial fibrosis in 12-week-old dystrophic muscle, but was significantly reduced in RAPA KO mice (Fig. 6a, b). In fact, ColVI staining was not significantly different between the RAPA LC and RAPA KO groups in the iliopsoas. Fibrosis is a key factor underlying mortality in muscular dystrophies because fibrotic accumulation in the diaphragm reduces its contractile ability and ultimately leads to respiratory failure. Importantly, we also observed a significant decrease in the fibrotic area of RAPA KO compared to VEH KO diaphragms (Fig. 6a, b), demonstrating the therapeutic potential of RAPA treatment for dystroglycanopathies.

### mTORC1 signaling correlates with levels of pathological markers in dystroglycanopathy muscle

**Fig. 7:**
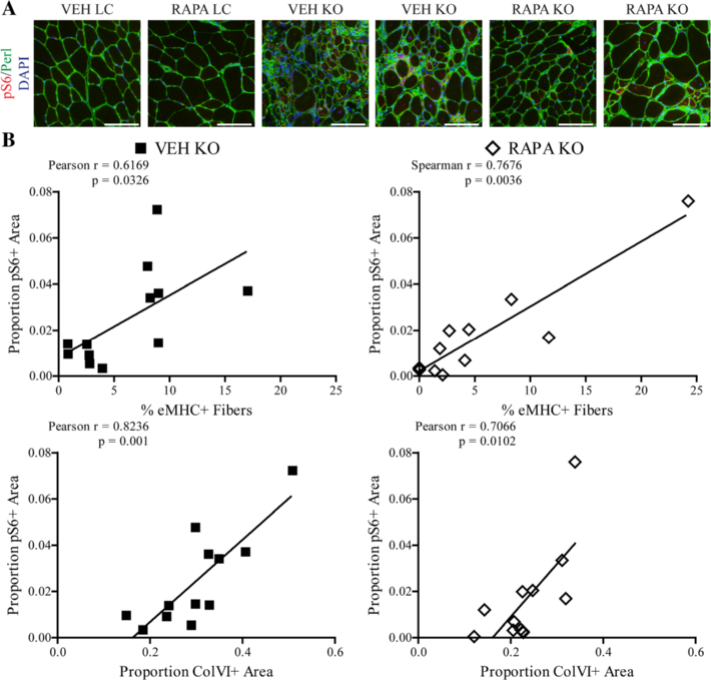
pS6 localizes to myofiber- and non-myofiber-specific niches in iliopsoas of Myf5/*Fktn* KO mice. **a** Images stained against phosphorylated S6 (S235/236) ribosomal protein (*red*) with basement membrane perlecan (*green*) and nuclear DAPI (*blue*) counterstains shown. Scale bar = 100 μm. **b** Plots comparing pS6-positive areas to percentages of regenerating fibers (eMHC, *top*) or to fibrotic areas (ColVI, *bottom*) in iliopsoas of VEH (*left*) and RAPA (*right*) KO mice. Pearson *r* value shown for all groups except RAPA KO eMHC (Spearman *r* shown, D’Agostino and Pearson omnibus normality test failed)

As mentioned previously, mTOR signaling has been linked to a number of muscle maintenance and disease processes, including regeneration and fibrosis. While the 4-week RAPA treatment improved histopathological features in treated Myf5/*Fktn* KO mice, its specific site of action is unclear. In particular, mTORC1 could be hyper-activated in only a subset of cells within the muscle compartment. Furthermore, because of ongoing regeneration and muscle remodeling in dystrophic tissue, the normal equilibrium of various resident cell types within the muscle niche likely changes as the disease state progresses. In order to evaluate whether a subset of cells and/or cell types demonstrate increased mTORC1 activity, we stained iliopsoas muscles from daily RAPA study mice with an antibody-detecting phosphorylation of S6 ribosomal protein, which is dependent on the mTOR substrate S6-kinase for activation. While levels of pS6 were below detection in LC mice, sections from KO mice revealed regions of robust staining (Fig. 7a). pS6 was observed in both the muscle fibers and endomysial spaces of KO iliopsoas, demonstrating that mTORC1 activation is not limited to a single cell type. Activated S6 was also observed in RAPA KO iliopsoas, as expected, because muscles analyzed were approximately 18 h to 2.5 days since the last VEH or RAPA dose for the muscle torque and treadmill RAPA studies, respectively. To delineate the potential locations of mTOR-mediated pathological processes, we correlated pS6 areas to markers of fibrosis (ColVI) or acute regeneration (eMHC). In VEH KO muscle, pS6 correlated weakly with eMHC levels (Pearson *r* = 0.6169) but tracked closely with levels of ColVI (Pearson *r* = 0.8236). In contrast, pS6 correlated more closely to eMHC (Spearman *r* = 0.7676) than to ColVI (Pearson *r* = 0.7066) in RAPA-treated KO muscle (Fig. 7b). Altogether, these results indicate a role for mTORC1 in both the regenerative and fibrotic processes of dystrophic muscle and suggest that RAPA may act to inhibit these signaling events in fibroblasts.

### Daily RAPA treatment partially reduces elevated autophagic flux in Fktn-deficient muscle

**Fig. 8:**
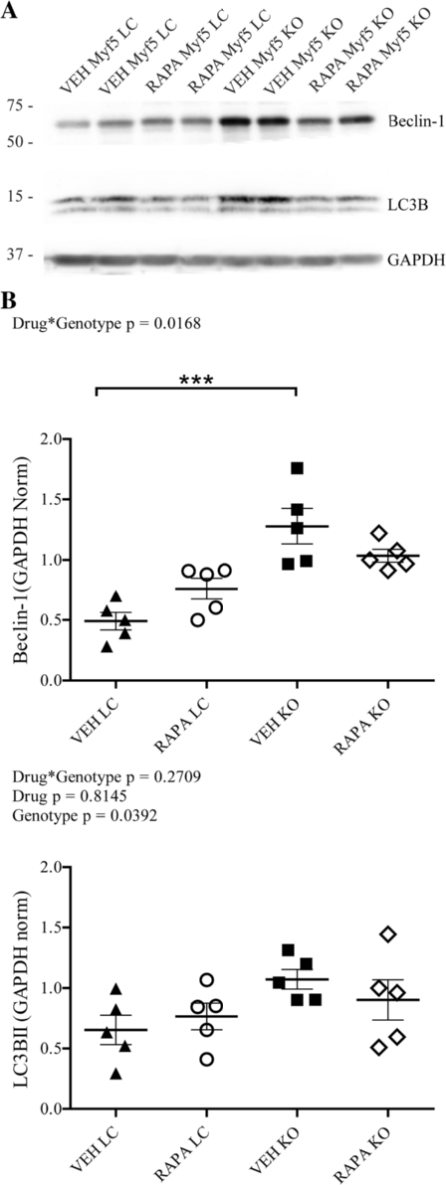
Autophagy proteins are upregulated in dystroglycanopathy muscle. **a** Western blot analysis showing levels of autophagy proteins Beclin-1 and LC3B. GAPDH was used as a loading control. **b** Densitometric analysis of Beclin-1 (*top*) and LC3B-II (*lower band*, *bottom*) protein levels, normalized to GAPDH loading control. Two-way ANOVA; ***p < 0.001

Recent work in mouse models has identified defective macroautophagy as a therapeutic target in dystrophic muscle [62, 63]; furthermore, pharmacologic or genetic reversal of this phenomenon, including by RAPA, appears to improve disease phenotype [33, 64, 65]. To probe levels of autophagy in *Fktn*-deficient muscle, we analyzed expression of the autophagosome-associated proteins Beclin-1 and LC3B by Western blot. We found a significant effect of genotype on relative quantities of the lipidated, autophagosome-partitioned form of LC3B (LC3B-II, lower band) (Fig. 8a, b). In addition, there was an interaction of drug and genotype on Beclin-1 levels with RAPA promoting an increase in littermate, but a decrease in KO mice; Beclin-1 was significantly elevated in VEH KO compared to VEH LC mice, in the post-test analysis (Fig. 8a, b). Western blot analysis of the autophagy regulator Vps15 also demonstrated a genotype effect, but the increased level of this protein in KO muscle was unaffected by RAPA treatment (data not shown). These data provide evidence of increased autophagy in dystroglycanopathy muscle, in contrast to reports in other models of muscular dystrophy. However, because autophagy functions to target defective proteins and organelles for cellular removal [66, 67], increased levels of Beclin-1 and LC3B-II in *Fktn-*deficient muscle likely point to clearance of tissue components following damage. For example, genes involved in mitochondrial biogenesis are upregulated following muscle injury as damaged organelles are replaced in the tissue [68]. Furthermore, in *mdx* muscle, pharmacologic modulation of 5′ adenosine monophosphate-activated protein kinase (AMPK) enhanced autophagy with an associated improvement of mitochondrial function and a coincident reduction of disease burden [64]. These data suggest a link between autophagy and mitochondrial remodeling in muscle. To determine whether RAPA treatment affects mitochondrial function, we assayed the activity of succinate dehydrogenase (SDH), a mitochondrial enzyme involved in oxidative phosphorylation at steps in both the citric acid cycle and the electron transport chain. We observed lower SDH activity in KO compared to LC muscle when normalized to total tissue mass but found no difference between RAPA and VEH KO mice. RAPA LC muscle tended to have more SDH activity than VEH LC muscle, but these differences were not statistically significant in the cohort tested (*n* = 4 RAPA LC, *n* = 3 VEH LC) (Additional file 4). Thus, RAPA-mediated improvements to Myf5/*Fktn* KO muscle appear to occur independently of mitochondrial function and further highlight the divergence of fukutin- and dystrophin-deficient muscle with respect to autophagic phenotype. Altogether, our results point to a myoprotective effect of RAPA treatment that may delay the progression of disease in dystroglycanopathy tissue.

## Discussion

Several muscular dystrophies arise from the loss of the structural linkage between the extracellular matrix and the intracellular cytoskeleton (reviewed in [69, 70]). While increased susceptibility of the sarcolemma to damage is a well-studied component of disease etiology, some signaling functions have also been ascribed to the DGC. Growth-receptor bound protein 2 (Grb2) has been shown to bind intracellularly to βDG, providing a possible link between the DGC and mTOR signaling through the extracellular signal-regulated kinase/ribosomal S6 kinase (ERK/RSK) pathway [71, 72]. Indeed, disruption of DG-laminin binding reduces ERK [73] and Akt activation and increases apoptosis in cultured myotubes [23]. However, consistent with the data presented here, a direct connection between αDG glycosylation and mTOR activation has not been demonstrated. Still, accumulating evidence suggests a critical role for mTOR signaling in the development and maintenance of the skeletal muscle [24, 53, 74–76].

Abnormal age- and muscle-dependent mTOR activation has been observed previously in the skeletal muscle of the *mdx* mouse, a mild model of dystrophin-deficient muscular dystrophy [30, 62, 77]. Furthermore, simultaneous increases in mTOR phosphorylation and protein content have also been described in *mdx* diaphragm at 10 weeks [64]. Here, we provide evidence of increased mTORC1 activation in aged *Fktn*-deficient muscle, a model of moderate to severe muscular dystrophy; however, changes in mTOR signaling were not intrinsic to the dystroglycan glycosylation defect as they did not arise until after the development of muscle pathology. Thus, activation of mTOR likely reflects the fibrotic progression that occurs during the dystrophic process. mTOR is indeed a known regulator of extracellular matrix protein synthesis in cultured fibroblasts, a process with apparent sensitivity to RAPA [78, 79], and is activated in primary muscle fibroblasts in response to growth stimuli [52]. It is therefore possible that the therapeutic benefit of RAPA treatment in dystroglycanopathy mice proceeds by impeding the development of endomysial fibrosis.

In agreement with our findings, mTORC1 inhibition has also shown therapeutic benefit in *mdx* mouse, although a consistent mechanism of action has not yet been established (e.g., [30, 33]). The outcomes reported in Fig. 3 seem to support a RAPA-mediated delay of disease progression in dystroglycanopathy muscle. Functional phenotypes of LC and KO mice are expected to diverge with the advancement of muscle disease, as was the case for in vivo torque measurements: VEH LC, but not VEH KO mice, demonstrated age-associated increases in torque following the 4-week dosing study; however, both RAPA LC and KO animals demonstrated an increase in torque at the conclusion of dosing. Analysis of downhill treadmill run distances between KO treatment groups shows a similar finding as four of seven RAPA-treated ran KO mice ran at least half the distance of their respective age- and treatment-matched controls, compared to a single instance in the VEH KO group. It is unclear why little to no improvement was observed in the remaining three RAPA KO mice, although dystrophic phenotypes of Myf5/*Fktn* KO mice have been reported to be variable [18]. If RAPA treatment is capable of delaying disease progression, but incapable of reversing dystrophy, then mice with more severe muscle involvement at the commencement of the dosing schedule might have a correspondingly weaker response to the drug.

Recent mechanistic studies have focused on the role of autophagy in both normal and dystrophic muscle, with the finding that consistent Akt or mTORC1 activation inhibits autophagic processes in a way that is damaging to muscle tissue [80]. Indeed, increasing evidence demonstrates that restoration or augmentation of autophagy can improve pathological features of muscle disease [33, 63, 64, 81]. Autophagy serves a key function in the clearance of damaged organelles from the cytosolic space, and accumulation of these dysfunctional cellular structures likely underpins the association between impaired autophagy and the dystrophic phenotype [63, 82]. Conversely, unchecked activation of the autophagy pathway is also detrimental to the muscle and leads to atrophy or myopathy [83, 84]. Here, we report that proteins involved in autophagosome formation are upregulated in dystroglycanopathy muscle, suggesting that autophagy is not deficient in dystroglycanopathy muscle (Fig. 8). However, we cannot distinguish between pathogenic deregulation of protein/organelle degradation pathways or an increase in cellular component recycling following repeated bouts of myonecrosis. In either case, RAPA appears to partially relieve the altered autophagic phenotype of Myf5/*Fktn* KO mice.

Rapamycin (sirolimus) and related rapalogs (everolimus, temsirolimus) are FDA-approved drugs for the treatment of tuberous sclerosis complex (TSC), lymphangioleiomyomatosis (sirolimus), renal transplant (sirolimus, everolimus), breast cancer, pancreatic cancer, subependymal giant astrocytoma (SEGA, everolimus), and renal cell carcinoma (everolimus, temsirolimus) [54]. Importantly, sirolimus and everolimus are in use as single and combination drug therapies in pediatric populations for TSC, TSC-related SEGA, and for children 13 years and up with renal transplant [85–89]. The 2-mg/kg RAPA dose used here, relatively low for mouse experiments, by allometric scaling is approximately equivalent to 5.7 mg/m^2^ [90]. In comparison, TSC and SEGA trials used doses as low as 1.5 or 3 mg/m^2^ (sirolimus and everolimus, respectively) and as high as ~4 or 4.5 mg/m^2^ (sirolimus, everolimus, respectively). In clinical trials evaluating sirolimus for renal transplant, a loading dose of 15 mg with a 5-mg maintenance dose (corresponding to 7.89 and 2.63 mg/m^2^, respectively, based on 1.9 m^2^ average male body area) was found to be safe. Thus, while the dose described in our study is at the higher range of dosage in pediatric patients, there is no evidence to support that this dose is too high to be considered clinically relevant. Potential adverse events, such as immunosuppression, metabolic changes, and hypertension, need to be considered; however, the clinical cohorts with adverse event reporting are predominately cancer or transplant patients, who consequently have significant comorbidities and combined therapies that may or may not be relevant to a dystroglycanopathy patient population [91]. Notably, seven children, ranging in age from 4 to 16 years old received rapamycin for intractable epilepsy due to tuberous sclerosis complex; daily treatment for 12 months was reported without significant side effects [86]. While an absence of liver and kidney toxicity in the fourth week of daily treatment in this study cannot exclude potential adverse events with longer RAPA treatment schedules in dystroglycanopathy mice or patients, previous work found that long-term RAPA treatment (estimated 2.2 mg/kg/day) increased lifespan of heterogeneous wild-type mice, with half of the study mice receiving therapy for approximately 75 weeks [92]. Therefore, while there is evidence that chronic RAPA treatment may be tolerable in mouse and human studies, caution is still warranted. Additional studies will be necessary to confirm the therapeutic value of long-term mTORC1 inhibition in dystroglycanopathy and to determine the optimal timing of treatment to ensure maximal tissue response.

## Conclusions

We found evidence of elevated mTOR activation in later-stage dystroglycanopathy muscle of *Fktn*-deficient mice. Younger mice treated for 4 weeks with RAPA displayed a reduction in fibrosis and muscle damage and had improved muscle function. We propose that RAPA may protect dystrophic muscle from damage to delay progression of disease and inhibit fibrotic remodeling. This suggests that mTORC1 signaling may be a viable therapeutic target for dystroglycanopathy-type muscular dystrophies, but further work is needed to define ideal treatment conditions.

## Additional files

Additional file 1: (A) Quantification of Akt or S6 protein expression in 17–25-week-old Myf5/*Fktn* LC and KO mice. Two-tailed Student’s *t* test. **p* < 0.05. (B) Akt expression has a significant correlation with the proportion of ColVI+ area in KO muscle (Pearson *r* = 0.7922, *p* = 0.0337). *n* = 8 Myf5/*Fktn* LC and 8 Myf5/*Fktn* KO mice (*n* = 7 per group for ColVI analysis due to tissue artifacts). (PDF 325 kb) Additional file 2: (A) Distribution of muscle fiber minimum diameter from non-regenerated muscle fibers in iliopsoas of VEH- and RAPA-treated LC and KO mice. Fibers are grouped into bins of 2.5 μm. Two-way ANOVA performed for each bin. *, Drug*Genotype *p* < 0.05; †, Genotype *p* < 0.05. *n* = 5 VEH LC (tissue artifact), *n* = 5 RAPA LC (tissue artifact), *n* = 7 VEH KO, *n* = 7 RAPA KO. (B) Distribution of muscle fiber minimum cross-sectional diameter from regenerated muscle fibers in iliopsoas of VEH- and RAPA-treated KO mice. Fibers are grouped into bins of 2.5 μm. Regenerated fiber numbers range from 64 to 360 in VEH KO mice and 5–217 in RAPA KO mice. *n* = 7 VEH KO, *n* = 6 RAPA KO (mouse sample with only five regenerating fibers was excluded from fiber size bin analysis). Two-tailed Student’s *t* test; **p* < 0.05. (PDF 276 kb) Additional file 3: (A) Fold-change over background values for pro-inflammatory cytokines (IL-1β, MCP-1, and TNF-α) tested via MILLIPLEX Map assay in quadriceps of daily RAPA study mice. (B) Fold-change over background values for TGF-β tested via MILLIPLEX Map assay in quadriceps muscle of daily RAPA study mice. (TIF 694 kb) Additional file 4: Tissue mass-normalized values of cytochrome C reduced in vitro by succinate dehydrogenase from homogenized TAs of VEH- or RAPA-treated LC and KO mice. Two-way ANOVA. (PDF 291 kb)

## Abbreviations

ANOVA: analysis of variance
CK: creatine kinase
CN: central nucleation
ColVI: collagen VI
DGC: dystrophin-glycoprotein complex
DMD: Duchenne muscular dystrophy
eMHC: embryonic myosin heavy chain
ERK: extracellular signal-regulated kinase
GAPDH: glyceraldehyde 3-phosphate dehydrogenase
Grb2: growth-receptor bound protein 2
H&E: hematoxylin and eosin
iKO: inducible knockout
KO: knockout
LC: littermate control
mTOR: mammalian target of rapamycin
mTORC1: mTOR complex 1
mTORC2: mTOR complex 2
Myf5: myogenic factor 5
PBS: phosphate-buffered saline
PDK1: phosphoinositide-dependent kinase 1
PDK2: phosphoinositide-dependent kinase 2
RAPA: rapamycin
RSK: ribosomal s6 kinase
SDH: succinate dehydrogenase
TA: tibialis anterior
Tam: tamoxifen
TGF-β: transforming growth factor-β
VEH: vehicle
αDG: α-dystroglycan
βDG: β-dystroglycan
