# Supplementary figures and images for: Four-week rapamycin treatment improves muscular dystrophy in a fukutin-deficient mouse model of dystroglycanopathy
Source: Skelet Muscle. 2016 Jun 2;6:20. doi: 10.1186/s13395-016-0091-9 (PMC4890530; doi:10.1186/s13395-016-0091-9)

**A****▲ LC    ■ KO**

Relative Expression

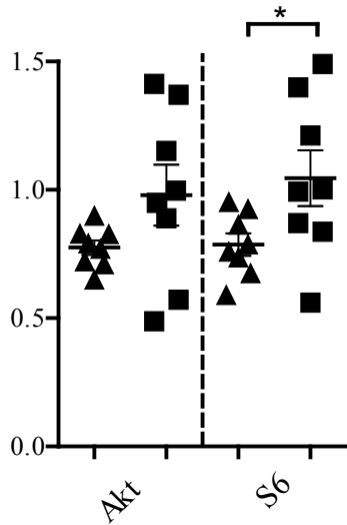**B**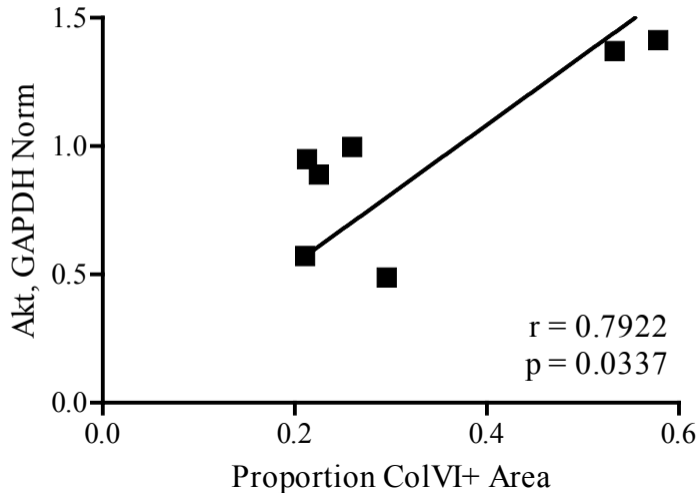

Supplement: Additional file 1: — (A) Quantification of Akt or S6 protein expression in 17–25-week-old Myf5/Fktn LC and KO mice. Two-tailed Student’s t test. *p < 0.05. (B) Akt expression has a significant correlation with the proportion of ColVI+ area in KO muscle (Pearson r = 0.7922, p = 0.0337). n = 8 Myf5/Fktn LC and 8 Myf5/Fktn KO mice (n = 7 per group for ColVI analysis due to tissue artifacts). (PDF 325 kb) [file 13395_2016_91_MOESM1_ESM.pdf]

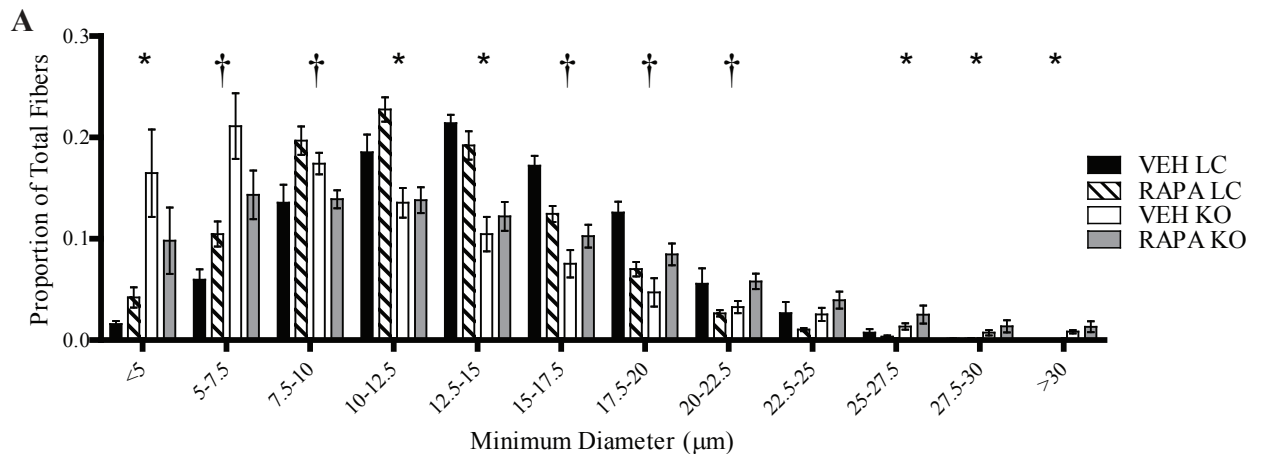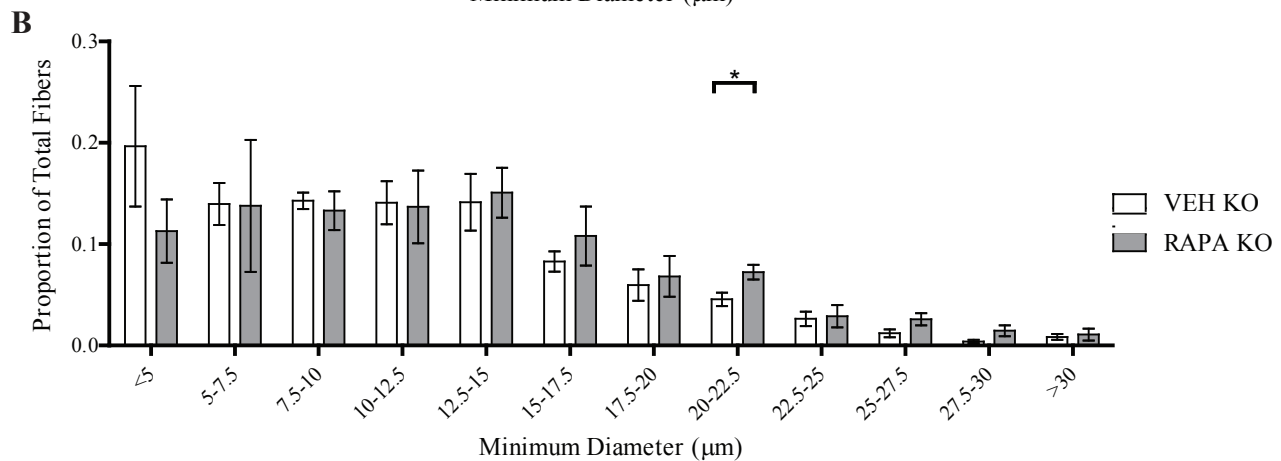

Supplement: Additional file 2: — (A) Distribution of muscle fiber minimum diameter from non-regenerated muscle fibers in iliopsoas of VEH- and RAPA-treated LC and KO mice. Fibers are grouped into bins of 2.5 μm. Two-way ANOVA performed for each bin. *, Drug*Genotype p < 0.05; †, Genotype p < 0.05. n = 5 VEH LC (tissue artifact), n = 5 RAPA LC (tissue artifact), n = 7 VEH KO, n = 7 RAPA KO. (B) Distribution of muscle fiber minimum cross-sectional diameter from regenerated muscle fibers in iliopsoas of VEH- and RAPA-treated KO mice. Fibers are grouped into bins of 2.5 μm. Regenerated fiber numbers range from 64 to 360 in VEH KO mice and 5–217 in RAPA KO mice. n = 7 VEH KO, n = 6 RAPA KO (mouse sample with only five regenerating fibers was excluded from fiber size bin analysis). Two-tailed Student’s t test; *p < 0.05. (PDF 276 kb) [file 13395_2016_91_MOESM2_ESM.pdf]

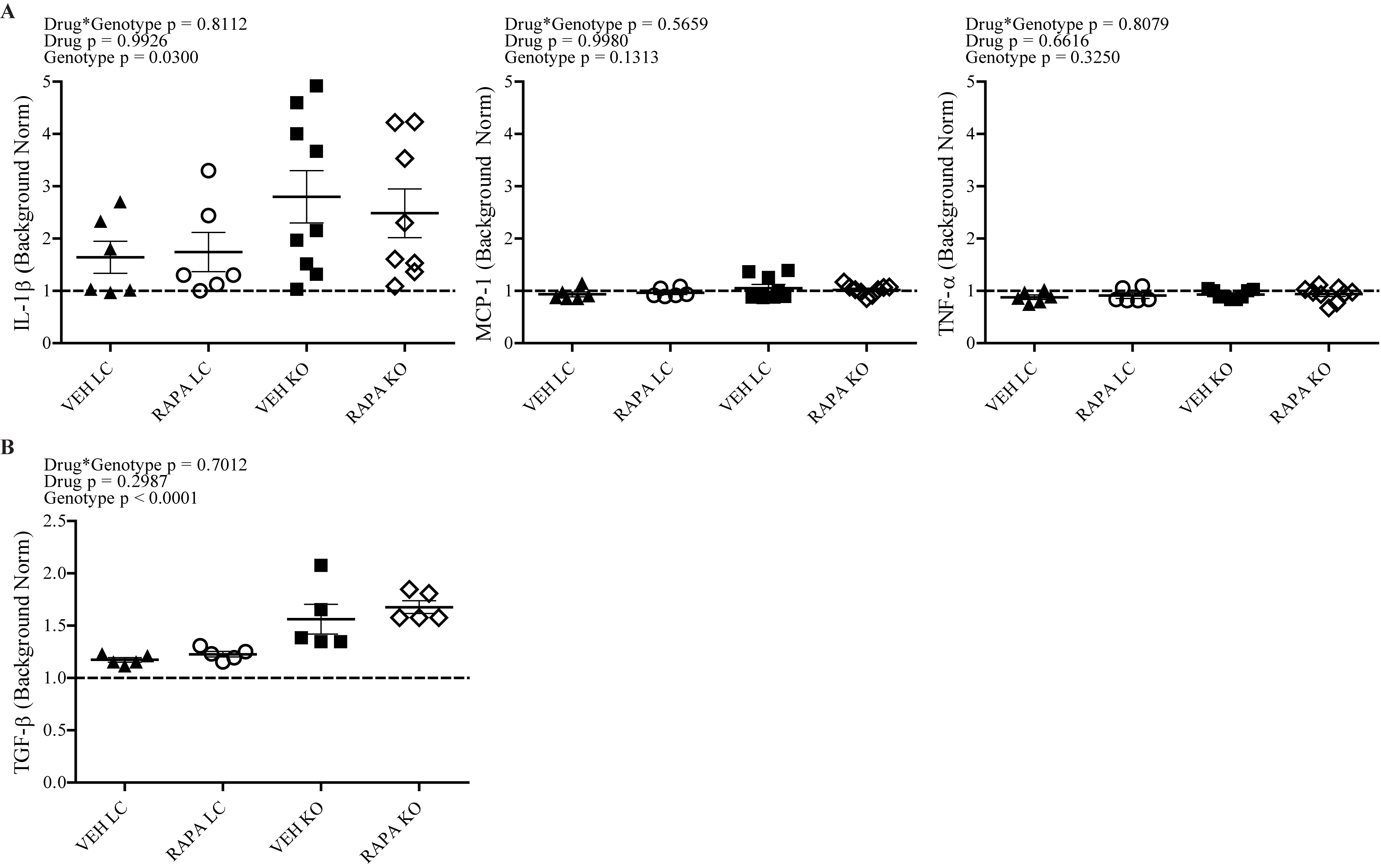

Supplement: Additional file 3: — (A) Fold-change over background values for pro-inflammatory cytokines (IL-1β, MCP-1, and TNF-α) tested via MILLIPLEX Map assay in quadriceps of daily RAPA study mice. (B) Fold-change over background values for TGF-β tested via MILLIPLEX Map assay in quadriceps muscle of daily RAPA study mice. (TIF 694 kb) [file 13395_2016_91_MOESM3_ESM.tif]

$\mu\text{mol cyt c/min/mg tissue mass}$

Drug\*Genotype  $p = 0.2643$   
Drug  $p = 0.1372$   
Genotype  $p = 0.0451$

VEH LC

RAPA LC

VEH KO

RAPA KO

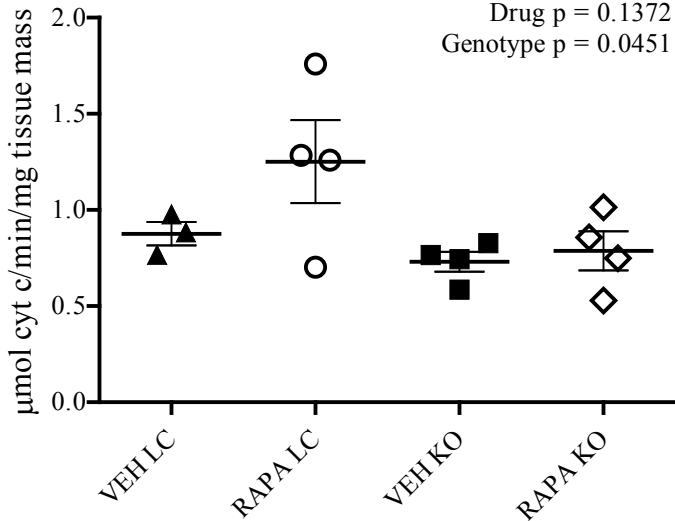

Supplement: Additional file 4: — Tissue mass-normalized values of cytochrome C reduced in vitro by succinate dehydrogenase from homogenized TAs of VEH- or RAPA-treated LC and KO mice. Two-way ANOVA. (PDF 291 kb) [file 13395_2016_91_MOESM4_ESM.pdf]
